# Detecting Pests From Light-Trapping Images Based on Improved YOLOv3 Model and Instance Augmentation

**DOI:** 10.3389/fpls.2022.939498

**Published:** 2022-07-07

**Authors:** Jiawei Lv, Wenyong Li, Mingyuan Fan, Tengfei Zheng, Zhankui Yang, Yaocong Chen, Guohuang He, Xinting Yang, Shuangyin Liu, Chuanheng Sun

**Affiliations:** ^1^National Engineering Research Center for Information Technology in Agriculture, Beijing, China; ^2^National Engineering Laboratory for Agri-product Quality Traceability, Beijing, China; ^3^College of Information Science and Technology, Zhongkai University of Agriculture and Engineering, Guangzhou, China

**Keywords:** pests and diseases, pest detection, YOLOv3, image cropping, convolutional neural network

## Abstract

Light traps have been widely used as effective tools to monitor multiple agricultural and forest insect pests simultaneously. However, the current detection methods of pests from light trapping images have several limitations, such as exhibiting extremely imbalanced class distribution, occlusion among multiple pest targets, and inter-species similarity. To address the problems, this study proposes an improved YOLOv3 model in combination with image enhancement to better detect crop pests in real agricultural environments. First, a dataset containing nine common maize pests is constructed after an image augmentation based on image cropping. Then, a linear transformation method is proposed to optimize the anchors generated by the k-means clustering algorithm, which can improve the matching accuracy between anchors and ground truths. In addition, two residual units are added to the second residual block of the original YOLOv3 network to obtain more information about the location of the underlying small targets, and one ResNet unit is used in the feature pyramid network structure to replace two DBL(Conv+BN+LeakyReLU) structures to enhance the reuse of pest features. Experiment results show that the mAP and mRecall of our proposed method are improved by 6.3% and 4.61%, respectively, compared with the original YOLOv3. The proposed method outperforms other state-of-the-art methods (SSD, Faster-rcnn, and YOLOv4), indicating that the proposed method achieves the best detection performance, which can provide an effective model for the realization of intelligent monitoring of maize pests.

## 1. Introduction

Maize is the staple crop with the largest production worldwide, with an estimated 1,026 million tons (Cerquiglini et al., 2016). This grain is the basic food for calorie and protein intake in developing countries (Shiferaw et al., 2011). However, the wide variety of pests in the field brings a great obstacle to corn production. Therefore, the control of common maize pests is of great importance for maize production (Fotso Kuate et al., 2019; Veres et al., 2020; Wang et al., 2020; Ratnadass et al., 2021). Traditionally, spraying chemical pesticides is still the primary way to implement the pest control task, however, inappropriate use of pesticides can damage the ecosystem and reduce the agricultural economy (Dhananjayan et al., 2020). In order to optimize the use of pesticides for periods and areas where pests are present, it is necessary to detect insect pests and evaluate their population.

Light traps have been widely used as effective tools to monitor multiple agricultural and forest insect pests simultaneously. However, for a long time, the recognition and counting of insects in these light traps have been mainly performed manually by humans, which is not only time-consuming and easy to make mistakes but also the delay of information acquisition will lead to missing the best time to pest control (Ding and Taylor, 2016). To avoid labor-intensive manual counting, image-based automated recognition methods have been widely reported in many studies (Fina et al., 2013; Xiao et al., 2018; Jiao et al., 2020; Wang et al., 2020). The image-based method captures images of traps and recognizes and detects pests on traps using image processing technology. Generally, these image-based methods include two main types: traditional machine learning-based methods and deep learning-based methods. The methods based on traditional machine learning rely on handcrafted features, which are usually designed for some specific tasks. For example, Fina et al. (2013) combined a k-means clustering algorithm with corresponding filters to achieve pest detection and identification. The detection method uses relative filters for feature extraction of different types of pests and provides effective identification of pests, but it is not very applicable in the case of a large amount of pest data and complex background. Liu et al. (2016a) used maximum stable extreme value region descriptors to simplify the background of field images containing aphids and then developed aphid recognition models using histograms of oriented gradient features and support vector machines, achieving an average recognition rate of 86.81% percent. Compared with methods based on traditional machine learning, methods based on deep learning have shown more robust and higher performance for the task such as pest classification and detection. For example, Liu et al. (2019) proposed a region-based end-to-end method PestNet and applied contextual RoI (Contextual Region of Interest) as contextual information of pest features to improve the accuracy of detection with good detection results in Multi-class Pest Dataset 2018 (MPD2018). One year later, Jiao et al. (2020) designed a CNN-based pest feature extraction module, and also introduced perceptual fields in the region proposal generation network, and changed the IoU-based matching method to construct an end-to-end two-stage framework that improves the detection accuracy of small pests. Successively, Liu and Wang (2020) designed a multi-scale feature detector using image pyramids to improve the detection accuracy and speed of tomato pests and diseases, and Zhang et al. (2020) introduced an improved Faster RCNN architecture using Online Hard Sample Mining Strategy in the training phase to enhance the detection of pests. A step further, to improve the performance of small target detection of pests, Lyu et al. (2021) proposed a feature fusion SSD algorithm based on Top-Down strategy, and Wang et al. (2021) introduced the attention mechanism into the residual network to obtain detailed pest characteristics and proposed an adaptive RoI selection method for pinpointing and classifying small pests.

However, the current pest detection methods still have some limitations, including: (1) Exhibit extremely imbalanced class distribution. A few classes contribute to most of the training samples, while some classes are under-represented in data. (2) Occlusion among multiple pest targets, a large number of redundant bounding boxes lead to low accuracy. (3) Inter-species similarity, fine-grained detection becomes more difficult.

In this study, to address these problems, we first collected a large number of very small-scale pest instances, including 17,049 images with nine categories. Second, we preprocessed the unbalanced original data by instance augmentation to reduce the impact of unbalanced sample distribution on model training, then we proposed a method to generate anchors based on k-means linear scaling to improve the matching of anchors with real boxes. Finally, we effectively used the residual structure to improve the reusability of features by the model and used a single detection head to reduce the computation of anchors during prediction. Through extensive experimental analysis, our proposed method outperforms other advanced detectors with better detection performance.

In summary, our main contributions to this study are listed as follows:

(1) The number of pest instances is balanced by instance expansion to reduce the impact of model training on pest classes with few instances.
(2) A k-means based linear transformation method was proposed to improve the matching of anchors to real labels.
(3) Changing the Backbone network and Head of the original YOLOv3 architecture to improve detection performance.
(4) The proposed method achieved an mAP of 77.29% and mRecall of 68.90%, which outperforms other advanced detectors.

## 2. Materials and Methods

### 2.1. Data Introduction

**Figure 1:**
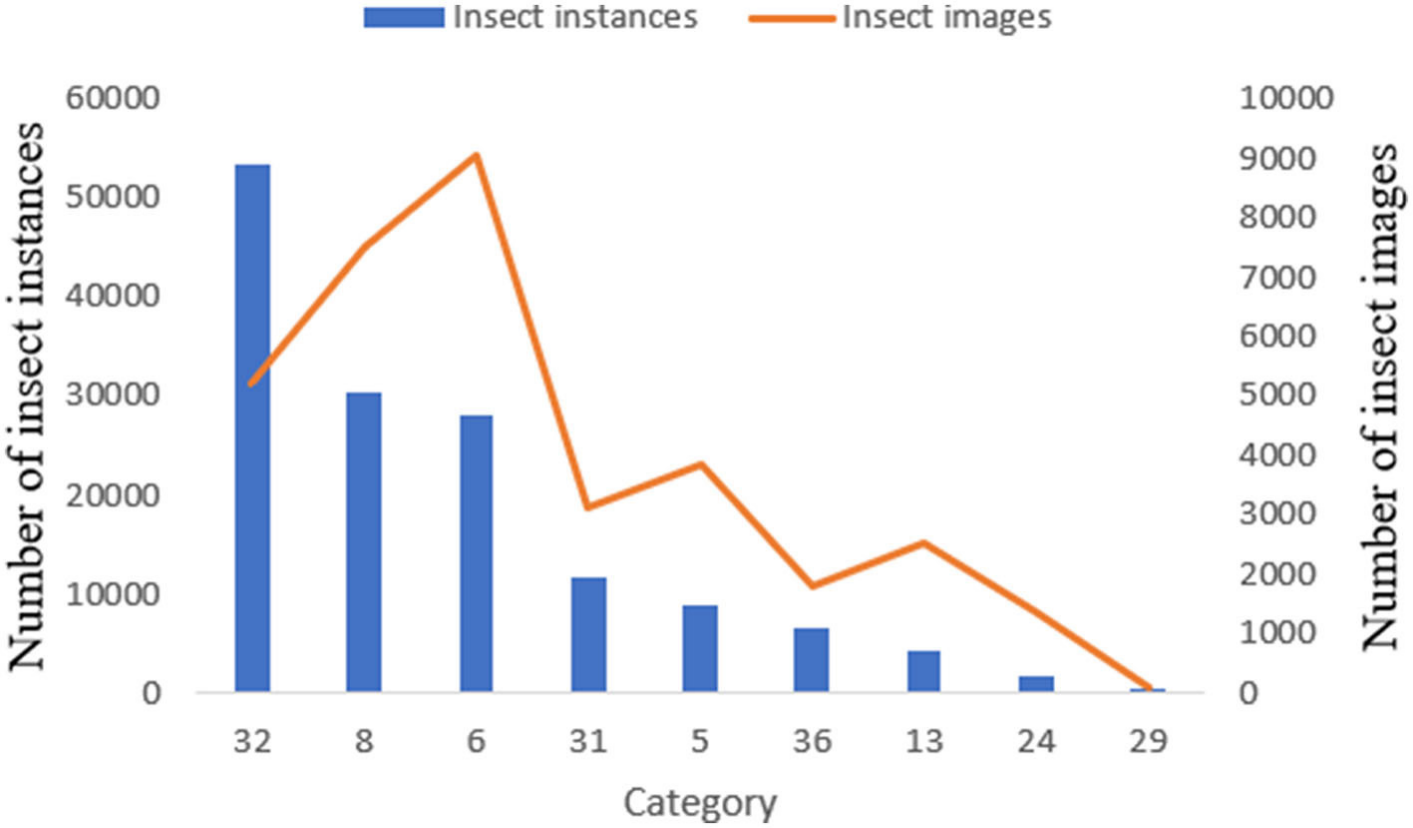
The number of instances and images for each pest category.

In this study, to construct a specific dataset and improve the detection accuracy for pest monitoring in the maize field, we selected nine common species of maize pest, namely Armyworm, Bollworm, Athetis Lepigone, Little Gecko, Yellow Tiger, Holotrichia Oblita, Holotrichia Parallela, Anomala Corpulenta, Agriotes Fuscicollis Miwa, as shown in Table 1. The image collection was done by collating from the public dataset **Pest24** (Wang et al., 2020), which is a large-scale multi-target standardized data set of light trap pests. During the dataset collation, we constructed a new dataset by traversing the operation to remove other kinds of tags from the XML file and also removed the images that have no target objects. The details of the collated datasets were shown in Table 1. We can see from Figure 1 that Holotrichia Oblita, Yellow Tiger has very few images and instances compared to the other seven classes of pests. Moreover, the existence of an overlap between pests reduces the accuracy of the model, such as the portrait of Athetis Lepigone in Table 1, and the similarity between pests such as Bollworm and Yellow Tiger is so similar that the model cannot extract features well, which leads to model overfitting.

**Table 1:** Description of the 9 classes of corn pests from the **Pest24** dataset.

**Pest name**	**Portrait**	**Index**	**Images**	**Instances**	**Images Augmentation**	**Instances Augmentation**	Average relativescale (%) ^*^
Armyworm	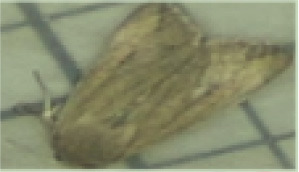	5	3,828	8,880	4,281	9,333	0.394
Bollworm	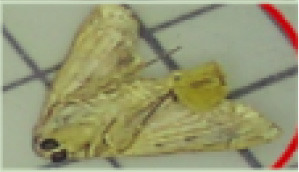	6	9,049	28,014	9,955	29,675	0.281
Athetis Lepigone	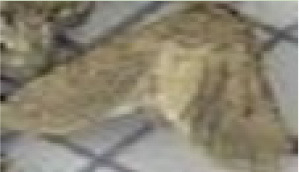	8	7,520	30,339	7,520	30,339	0.13
Little Gecko	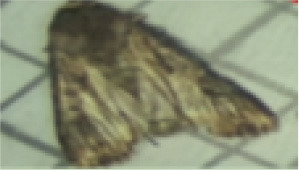	13	2,503	4,279	3,860	17,849	0.57
Yellow Tiger	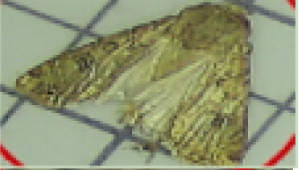	24	1,388	1,686	1,991	9,084	0.398
Holotrichia Oblita	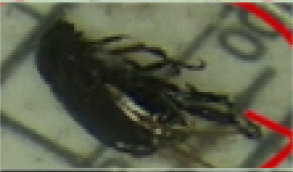	29	90	108	1,447	13,678	0.334
Holotrichia Parallela	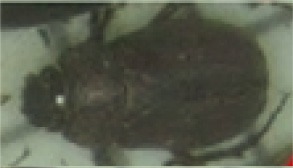	31	3,111	11,675	3,261	11,825	0.255
Anomala Corpulenta	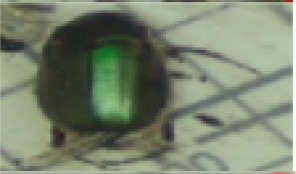	32	5,228	53,347	5,681	53,951	0.249
Agriotes Fuscicollis Miwa	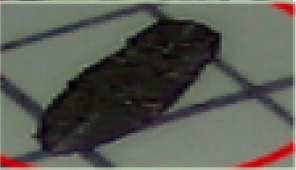	36	1,814	6,484	2,720	11,618	0.114

* *Represents (size of GT)/(size of original image)*.

In summary, the dataset is featured typically with extremely imbalanced class distribution, occluded distribution of insects, and high insect similarity. These features pose great challenges for object detection methods on the dataset.

### 2.2. Image Processing

#### 2.2.1. Image Enhancement

**Figure 2:**
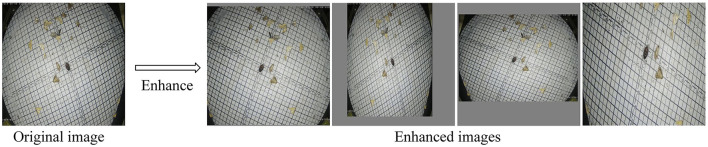
Original image and Enhanced images, where Enhanced images are images generated from different batches of original images after image enhancement operations. The Enhanced images in the figure represent the four images generated under four batches.

The image enhancement of training samples can improve the quality and diversity of samples, which is conducive to the improvement of CNN detection accuracy (Wan and Goudos, 2020). Data augmentation consists of two main categories, such as offline augmentation and online augmentation. Offline enhancement operates directly on the dataset and can be applied to relatively small datasets. For large datasets, online enhancement is a more appropriate approach. The essence of image enhancement is all operations such as spatial geometric transformation, pixel color transformation, and blurring of the original image. In this study, due to the large dataset, we used online enhancements. We scaled and distorted the aspect of the pest images of each batch during training data, added gray bars to the excess parts of the images, and flipped the scaling so that different batches have different input images, as shown in Figure 2, which can effectively improve the robustness of the model.

#### 2.2.2. Instance Augmentation

**Figure 3:**
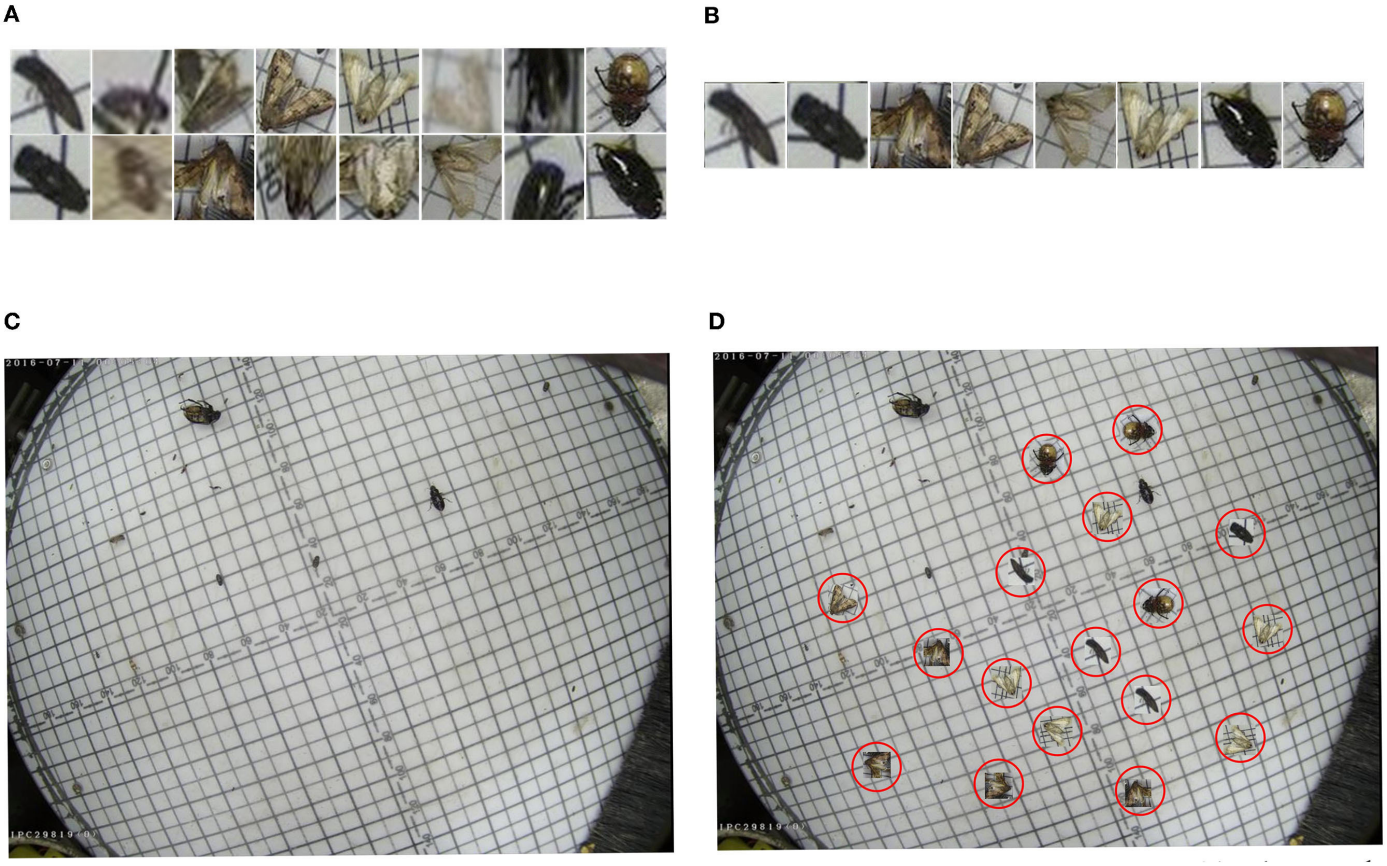
Schematic diagram of instance augumentation. **(A)** Iterate through all XML files and crop out instances of specific species of pests. **(B)** Manual selection to filter blurry and sticky images. **(C)** Select an image with a low number of pets from the dataset and use it as a background image. **(D)** Copy image instances to the selected background image.

To alleviate the problem of extremely imbalanced class distribution, instance expansion for pest species with few individuals was implemented in this step. We augmented the instances of specific pests by cropping and pasting and generating new images and xml files as shown in Figure 3.

**Figure 4:**
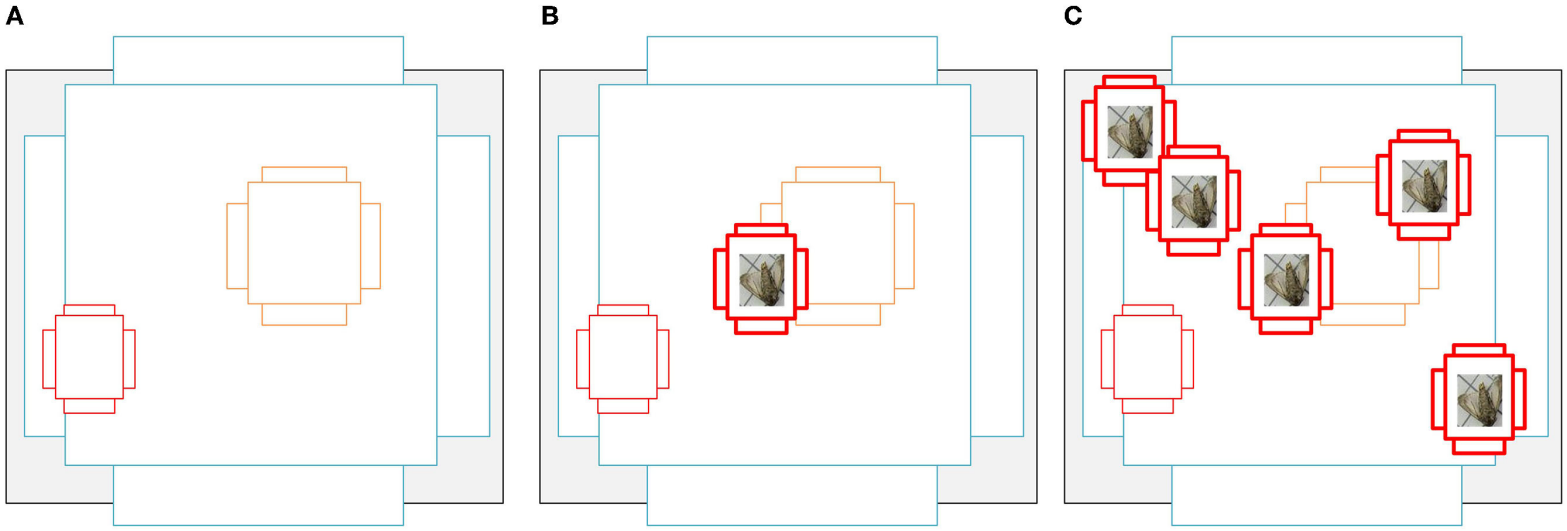
**(A)** is a schematic diagram of an anchor, and **(B)** represents one anchor point corresponding to a small target, there are only three anchors that can be paired with small targets, and the IoU of the pairing is not high. **(C)** originally had only one small target, and the number of anchors corresponding to it was three, but now it is copied in three copies, then there are four small targets in the graph, and the number of anchors corresponding to it becomes 15, which greatly increases the probability of this small target being detected.

We flipped the pests randomly before pasting them into the background image and ensured that the pasted objects did not overlap with any existing objects and that the pasted objects were as far away from each other as possible. Table 1 shows the number of pest instances and images after instance augmentation, and Figure 4 shows the matching of anchors with pests at different scales, it is clear that the number of matching anchors with pests is found to increase positively with the amount of pest paste, and the increase in anchor frames also improves the model's ability to detect small-sized pests and overlapping pests. We also divided the data for the expanded training, keeping the test set unchanged, the ratio of the training set to the validation set was 8:2. The divided training set, validation set, and test set were 12,465, 2,985, and 3,411 images, respectively, totaling 18,861 images.

### 2.3. Detection Method

**Figure 5:**
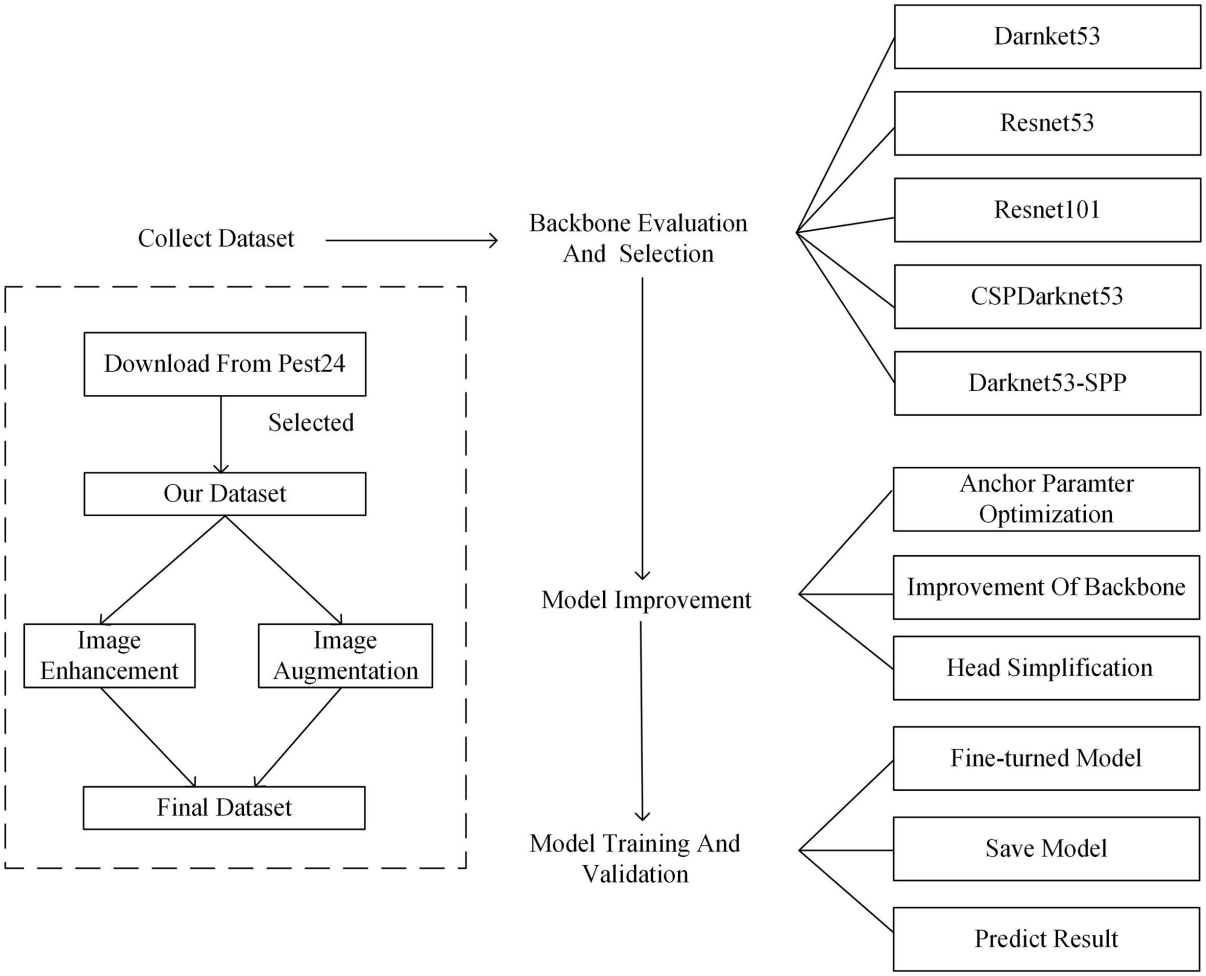
The overall process of the proposed model from (1) data collection to, (2) backbone selection and model improvement, and (3) model training and validation.

The whole flowchart of the proposed detection framework is shown in Figure 5. This flowchart mainly includes four stages: dataset collection, backbone evaluation and selection, model improvement, model training and validation. The collected data are input to the feature extraction network after image processing, and different backbone networks are used to extract features from the pest images, while the network structure is further optimized and trained to obtain the optimal model.

#### 2.3.1. YOLOv3 and Problem Analysis

The YOLO series model is one of the most commonly used target detection models, of which YOLOv3 is an improved version of YOLOv2. Compared with other commonly used models such as Faster-rcnn and SSD, YOLOv3 achieves a balance between detection time and accuracy (Adarsh et al., 2020), and has achieved great success in major fields such as military (Calderón et al., 2020) and pedestrian detection (Molchanov et al., 2017; Lan et al., 2018), and its most important feature is that it uses a feature pyramid network to achieve the fusion of multi-layer features, which improves the feature extraction capability of the network model. The feature extraction network of YOLOv3 uses the Darknet-53 network, which has a unique residual module to improve the stability and convergence speed of the detection model. In addition, YOLOv3 adopts a feature pyramid to achieve the fusion of features at different scales. Specifically, YOLOv3 downsamples the input image five times and considering that large-scale features are rich in image texture and grayscale information and small-scale features are rich in semantic information, YOLOv3 inputs the last three layers of the network into the feature pyramid to achieve the fusion of large-scale and small-scale feature information. YOLOv3 improves the feature extraction ability of the model and the accuracy of target detection and enhances the small target detection ability (Redmon and Farhadi, 2018).

In YOLOv3, the idea of anchors is borrowed from Faster-rcnn. The authors (Redmon and Farhadi, 2018) clustered the COCO dataset by k-means and generated nine anchors, such as (30×61), (62×45), (59×119), (30×61), (62×45), (59×119), (116×90), (156×198), and (373×326). Different anchors match different feature layers in three branches, each feature layer corresponds to a different receptive field. Thirty-two times downsampling has the largest receptive field, which is suitable for detecting large targets, so when the input is 416×416, the three anchor boxes for each cell are (116×90), (156×198), and (373×326). Sixteen times downsampling is suitable for the middle size object, the anchor boxes are (30×61), (62×45), and (59×119). Eight times downsampling has the smallest receptive field and is suitable for detecting small targets, so the anchor boxes are (10×13), (16×30), and (33×23). However, anchors generated by k-means can produce bad results in practical applications. Compared with the COCO dataset, most target detection datasets belong to specific scenarios where the real labels are only at a single or specific scale. Using k-means to generate anchors would result in objects of similar size being forced into different feature layers for prediction, which is obviously unreasonable. Therefore, we propose the following improvement methods.

#### 2.3.2. The Improved YOLOv3 Network

**Figure 6:**
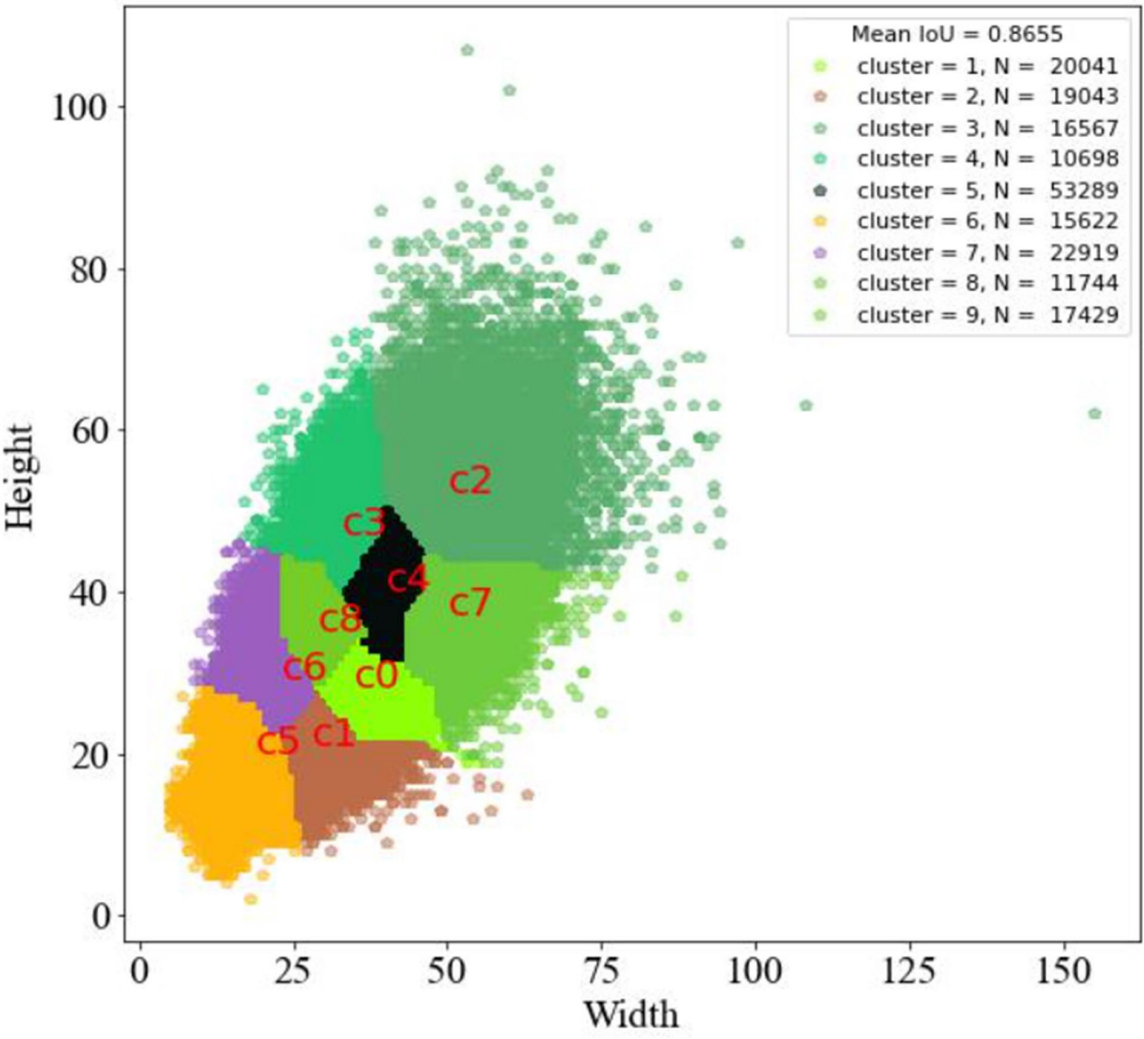
Size distribution and the number of pests. The kmeans clustering generates 9 centroids, cluster represents the centroid number, N represents the number of instances in each class, a total of 187,352.

When using the pest dataset for training, the anchor boxes generated by the k-means clustering algorithm are very concentrated as shown in Figure 6 because of the many types of pest images and the similar and concentrated size of the real labels, which do not reflect the advantage of the model's multi-scale output; Also, there exist many real boxes with larger sizes than the anchor boxes obtained by the k-means clustering algorithm, which is not conducive to the training of the model for pest localization.

##### 2.3.2.1. Anchor Parameter Optimization

**Figure 7:**
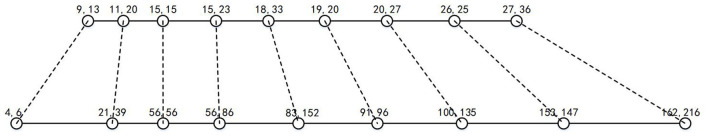
Schematic diagram of the linear shift of anchors.

To improve the matching of anchors to real labels, We propose a method to linearly scale the anchors by stretching the size of the anchor generated by the k-means algorithm to both sides to better fit the real frame at different scales of the pest dataset and improve the detection accuracy. The formula is as follows (1), (2), and (3).


(1) $$\begin{array}{l} {x_{9}}^{\prime }=\beta x_{9} \end{array}$$

(2) $$\begin{array}{l} {x_{i}}^{\prime }=\frac{\left(x_{i}-x_{1}\right)}{\left(x_{9}-x_{1}\right)}\left({x_{9}}^{\prime }-{x_{1}}^{\prime }\right)+{x_{1}}^{\prime } \end{array}$$

(3) $$\begin{array}{l} {y_{i}}^{\prime }={x_{i}}^{\prime }\frac{y_{i}}{x_{i}} \end{array}$$

where x_i_, y_i_ (i = 2, 3, ..., 9) represents the anchor with the serial number i before and after the transformation, the first anchor box is scaled by 0.5 times by default, β represents the stretch factor from the second anchor box to the ninth anchor box. The transformed schematic is shown in Figure 7.

##### 2.3.2.2. Improvement of Backbone

**Figure 8:**
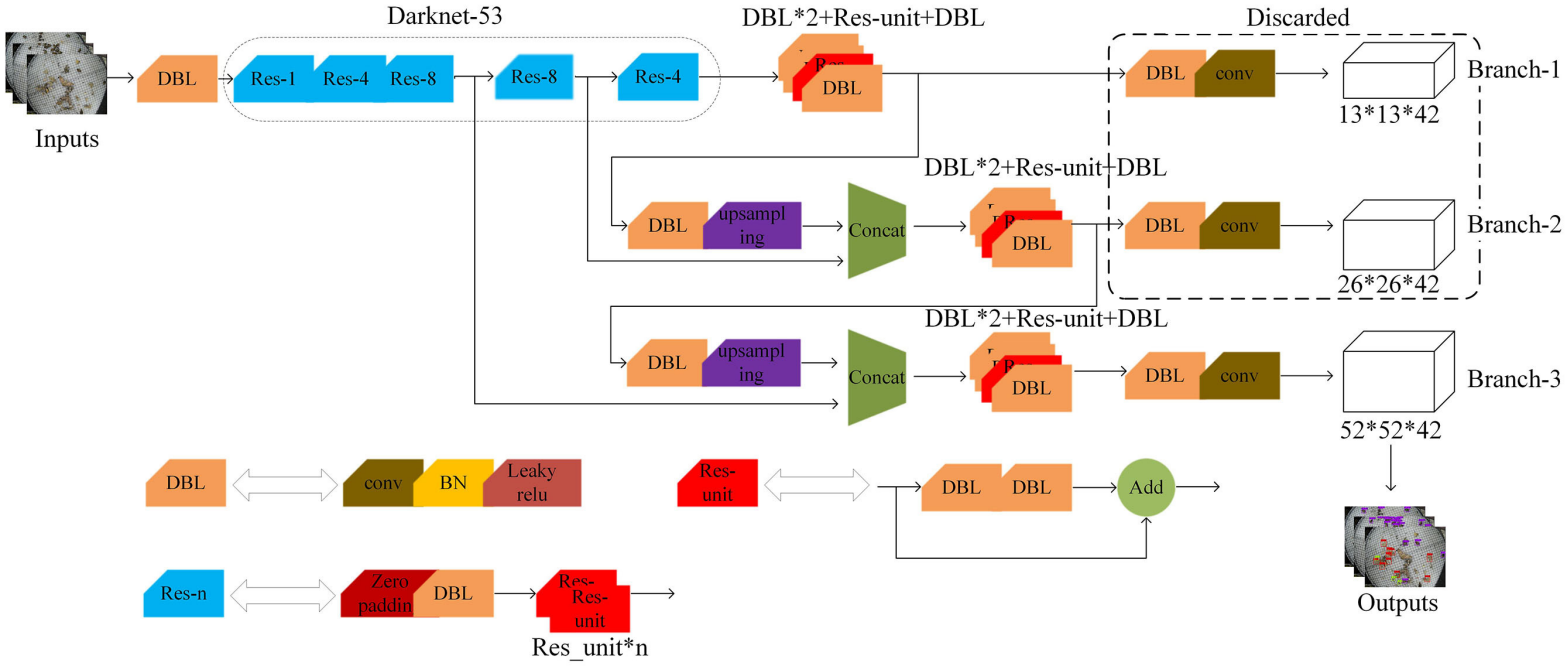
The architecture diagram of the improved YOLOv3 network.

Although YOLOv3 outputs a three-scale feature map through feature fusion and multi-objective prediction, the accuracy of pest detection still needs to be improved. The object detection output layer of the YOLO V3 network contains six DBL units and one 1×1 conv. Inspired by the DSSD network Fu et al. (2020), the first five DBL units are turned into three DBL units and one ResNet unit in order to avoid gradient disappearance and enhance feature reuse. Meanwhile, as shown in Figure 8, two residual units are added to the second residual block of the original network to extract more information about the location of small targets at lower levels. Backbone network input, convolution, and output details are shown in Table 2, Improved network makes extensive use of hopping connections of residuals and directly discards pooling in order to reduce the negative effect of gradients from pooling and uses conv to achieve downsampling. In this network structure, convolution with a step size of two is used for downsampling.

**Table 2:** Backbone architecture details.

	**Layer**	**Filter**	**Size**	**Output**
	Conv2	32	3× 3	256× 256
	Conv2	64	3× 3/2	128× 128
	Conv2	32	1× 1	
1×	Conv2	64	3× 3	
	Residual			128× 128
	Conv2	128	3× 3/2	64× 64
	Conv2	64	1× 1	
4×	Conv2	128	3× 3	
	Residual			64× 64
	Conv2	256	3× 3/2	32× 32
	Conv2	128	1× 1	
8×	Conv2	256	3× 3	
	Residual			32× 32
	Conv2	512	3× 3/2	16× 16
	Conv2	256	1× 1	
8×	Conv2	512	3× 3	
	Residual			16× 16
	Conv2	1,024	3× 3/2	8× 8
	Conv2	512	1× 1	
4×	Conv2	1,024	3× 3	
	Residual			8× 8
	Conv2	1,024	3× 3/2	8× 8

## 3. Experiment Design

### 3.1. Experimental Running Environment

Experimental platform: All experiments are performed on two GTX 2080Ti GPU with 11 GB memory, and the software is Window 10, Python 3.8, and CUDA 11.4. Pytorch and Caffe are used to build convolutional neural networks. Evaluation metrics: To fairly evaluate the detection performance, we use the standard average precision (AP) of each class and mean average precision (mAP) values with IoU thresholds at 0.5. Training settings: The models are trained with 2 GPU with a batch size of 16 for 100 epochs using the Adaptive Moment Estimation (Adam) optimizer. The learning rate is initialized to 0.002 and multiplied by 0.94 after each epoch.

### 3.2. Model Training

**Figure 9:**
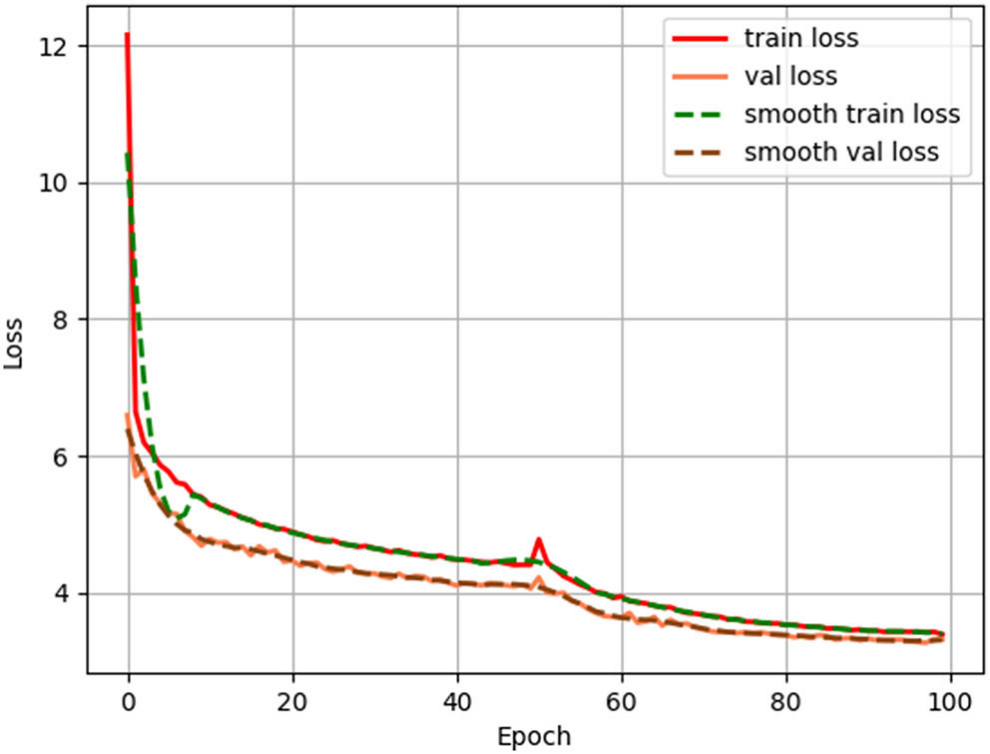
Loss and validation curves during the training process.

In this experiment, a transfer learning approach is used for training. The transfer learning uses the existing part of pre-training weights, and the part of the network to which these pre-training weights are applied is generic, such as Darknet53.We first freeze the training of this part of the weights and put more resources into training the later part of the network parameters, so that both time and resource utilization can be greatly improved. The later network parameters are trained for 50 epochs before the backbone network is unfrozen, and then all of them are trained together. This operation can accelerate the convergence of the model and reduce the training time of the model. The loss value curves of the training set and validation set of the improved model in this article are shown in Figure 9.

### 3.3. Model Evaluation Indices

Model evaluation is a key step in evaluating model detection performance and is the main criterion for verifying model robustness and detection capability. The commonly used indices for evaluating target detection models are Recall (R), Average precision (AP), Average category precision (mAP), and FPS, where the formulas for P, R, AP, and mAP are shown in Equations (4–7).


(4) $$\begin{array}{l} P=\frac{TP}{TP+FP} \end{array}$$

(5) $$\begin{array}{l} R=\frac{TP}{TP+FN} \end{array}$$

(6) $$\begin{array}{l} \begin{array}{c} AP=\int_{0}^{1}P(R)dR \end{array} \end{array}$$

(7) $$\begin{array}{l} \begin{array}{c} mAP=\frac{\int_{q=1}^{Q}AP(q)}{Q} \end{array} \end{array}$$

TP: Positive samples are correctly identified as positive samples.

FP: False positive samples, negative samples are incorrectly identified as positive.

FN: False negative samples, positive samples are incorrectly identified as negative samples.

P: The percentage of True positives among the recognized images. That is the proportion of all identified pests that are true positives in this hypothesis.

R: The ratio of all positive samples in the test set that are correctly identified as positive samples. In this hypothesis, the ratio of the number of correctly identified pests of a certain species to the number of all true pests of that species in the test set.

AP: The area under the precision-recall curve, generally the better a classifier is, the higher the AP value.

mAP: The average of all categories of AP represents a composite measure of the average accuracy of the detected targets.

FPS: Number of picture frames detected per second.

### 3.4. Experimental Results and Analysis

#### 3.4.1. Detection Performance With Different Stretch Factors

In order to improve the level of matching of anchors to real boxes, based on kmeans clustering to generate anchors, we perform a linear shift on the anchor to improve the performance of the model for pest detection. The maximum anchor enlargement factor is β. As can be seen from the figure, the map reaches a maximum of 77.19% when β is 7.

**Figure 10:**
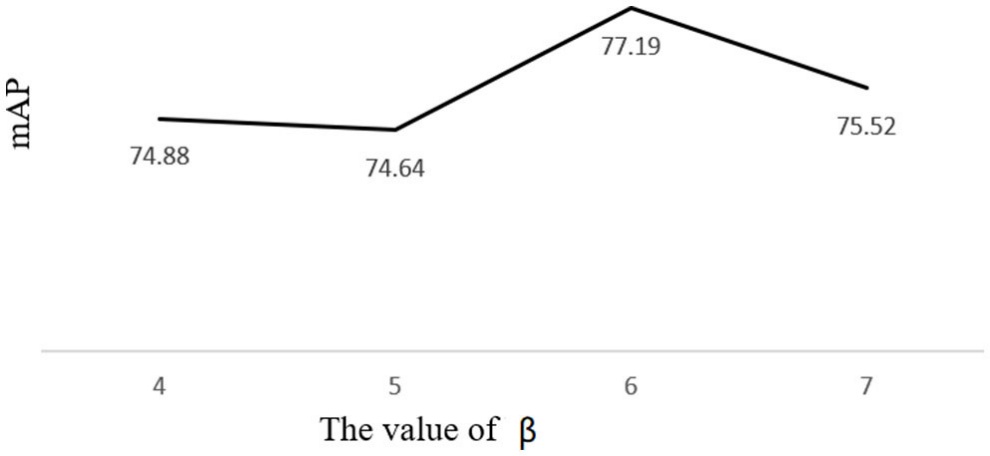
Schematic diagram of the linear shift of anchors.

In Figure 10, the folded graph shows that the anchors generated by the k-means algorithm do not match the real labels of the model well, resulting in poor model training and a low map effect. We propose a linear transformation to optimize the anchors, and the model has the best effect on pest detection with 77.19% when the anchor boxes are (4×6), (21×39), (56×56), (56×86), (83×152), (91×96), (100×135), (153×147), and (162×216).

#### 3.4.2. Detection Performance With Different Backbones

To evaluate the performance, we enhanced the proposed network further on the basis of the optimized anchor, compared with several excellent CNN networks, such as Resnet53, Resnet101, and CSPdarknet53, and optimized the Neck and Head of the model to derive the best detection model, which was configured to use the same configuration, such as Optimizer (Adam), weight decay (0.00005), the classifier (softmax) and learning rate (0.0002), gamma (0.94).

Table 3 shows the performance improvement of our backbone compared to other backbones. Among them, for all types of pests, the AP and R of our method are higher than those of other methods. Not only our method improves the detection performance for objects of different sizes but also reduces the rate of missed detection by the model for pests that need to be detected.

**Table 3:** Performance comparison results using different feature extraction and fusion methods.

**Classes**	**Darknet53**		**Resnet53**		**Resnet101**		**CSPDarknet53**		**Darknet53-SPP**		**Ours**	
	**AP**	**R**	**AP**	**R**	**AP**	**R**	**AP**	**R**	**AP**	**R**	**AP**	**R**
5	77.02	72.61	49.66	28.98	55.98	36.81	77.21	70.70	65.24	52.99	**79.87**	**74.14**
6	88.98	81.75	77.76	64.94	80.40	64.25	88.90	81.94	84.42	72.24	**90.02**	**84.31**
8	67.12	43.56	48.36	24.74	49.95	22.30	67.37	49.83	58.13	32.96	**69.63**	**48.56**
13	86.63	74.92	64.49	34.34	70.58	44.24	86.00	77.83	78.97	58.99	**86.82**	**78.58**
24	48.44	42.42	0.40	0.30	5.68	0.91	47.12	40.61	29.04	15.45	**54.18**	**48.48**
29	**59.41**	21.62	8.78	2.70	10.18	2.70	44.96	16.22	6.05	2.70	47.07	**27.03**
31	93.11	88.83	88.13	81.46	87.63	80.28	93.23	90.53	88.49	86.13	**93.42**	**92.19**
32	97.09	**96.41**	95.89	93.34	95.80	92.21	96.76	95.68	95.51	91.77	**97.09**	5.69
36	76.89	66.28	60.35	44.43	61.30	48.19	75.55	68.09	67.01	54.70	**77.46**	**71.13**

*The parts in bold represent the best performance*.

#### 3.4.3. Detection Performance With Different Head Branches

To further improve the inference speed of the model, we test the Head part of the model and detect the pest images to compare the FPS values before and after the model improvement, and the experimental results are shown in Table 4. It can be seen that among the three branches, Branch-3 plays a decisive role in the detection, while Branch-1 and Branch-2 do not play a detective effect. For this reason, we remove them in detection to reduce the corrective computation of prediction frames and true frames and improve the inference speed of the model.

**Table 4:** The detection performance of different branches.

**Method**	**Branch**	**Feature map**	**mAP**	**R**
Yolov3(baselines)	3 Branches	-	70.98	64.29
Ours	Branch-1	13× 13× 42	0	0
	Branch-2	26× 26× 42	0.09	0
	Branch-3	52× 52× 42	77.29	68.9
	3 Branches	-	77.29	68.9

#### 3.4.4. Ablation Experiments

We can see in Table 5 through the ablation comparison that our improved YOLOv3 model achieves good results in terms of detection accuracy and detection time and has good generalization performance for field pest datasets.

**Table 5:** Enhancement effect of different improvement methods.

	**Default**	**Instances augmentation**	**Anchor parameter Optimization**	**Improvement of backbone**	**mAP**	**R**	**FPS**
YOLOv3	✓				70.98	64.29	53
	✓	✓			73.52	60.49	54
	✓	✓	✓		77.19	65.37	54
Ours	✓	✓	✓	✓	**77.29**	**68.90**	**61**

*The parts in bold represent the best performance*.

#### 3.4.5. Comparison With Other Advanced Architectures

To validate the overall performance of the proposed method for pest detection, we compared it with state-of-the-art detectors, including the one-stage detectors SSD (Liu et al., 2016b), YOLOv3 (Redmon and Farhadi, 2018), YOLOv4 (Bochkovskiy et al., 2020), two-stage detectors Faster-RCNN (Ren et al., 2015). Table 6 shows the detection results of different models in our dataset. Compared with the original YOLOv3, our proposed method has improved mAP and R by 6.3 and 4.61%, respectively. The highest mAP and R of our method compared with other advanced methods indicates that our proposed method achieves the best detection results.

**Table 6:** Comparison of detection results of state-of-the-art detectors.

**Classes**	**YOLOv3**		**Faster-rcnn**		**SSD**		**YOLOv4**		**Ours**	
	**AP**	**R**	**AP**	**R**	**AP**	**R**	**AP**	**R**	**AP**	**R**
5	78.70	68.96	50.84	53.68	77.02	64.30	74.48	62.72	**79.87**	**74.14**
6	89.88	81.59	59.69	68.38	87.50	73.50	88.62	80.31	**90.02**	**84.31**
8	68.80	83.24	27.13	47.26	54.72	19.94	66.20	42.56	**69.63**	**48.56**
13	85.49	72.34	65.45	75.46	86.10	77.72	84.19	73.74	**86.82**	**78.58**
24	34.65	17.27	13.95	0.30	52.42	39.39	41.50	33.64	**54.18**	**48.48**
29	14.72	0.0	9.58	0	32.31	0.00	31.04	8.11	**47.07**	**27.03**
31	93.33	89.79	65.19	69.94	90.83	77.09	91.78	86.47	**93.42**	**92.19**
32	96.95	96.14	78.65	83.64	96.02	87.79	97.12	95.83	**97.09**	**95.69**
36	76.26	69.32	33.44	41.39	65.15	30.82	75.85	65.34	**77.46**	**71.13**
Mean	70.98	64.29	44.88	48.89	71.34	55.28	72.31	60.97	**77.28**	**68.90**

*The parts in bold represent the best performance*.

Table 7 shows the inference time and the total number of trainable parameters for the above methods. SSD has the least number of trainable parameters and the fastest inference speed. Followed by Faster-rcnn with the slowest inference speed, and finally, our proposed method, although it has the most trainable parameters, has the highest detection accuracy and the inference speed can reach the level of real-time application, 61 frames per second.

**Table 7:** Training parameters and inference time for different models.

**Model**	**Trainable parameters**	**Forward/backward pass size (MB)**	**Inference time(FPS)**
YOLOv3	61,566,814	992.36	54
Faster-rcnn	28,357,288	916.44	26
SSD	24,681,542	790.90	70
Ours	61,731,422	1117.79	61

**Figure 11:**
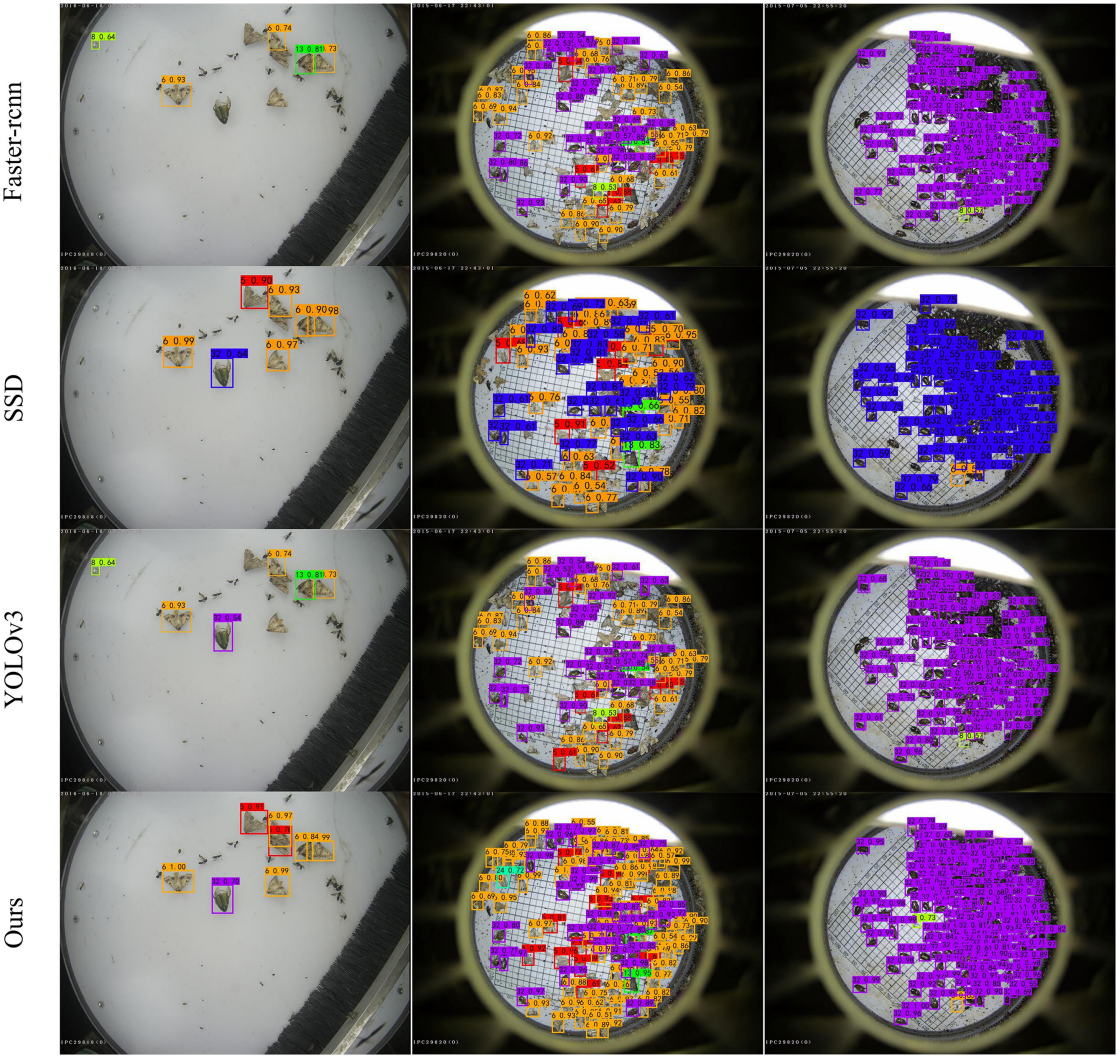
Examples of detection results using the proposed method and other state-of-the-art methods.

Our method performs well for sparse or dense pest instances. In particular, our method can accurately distinguish pests with similar textures and can detect and identify pests that overlap together with high performance as well. For example, as shown in Figure 11, in the first column of plots, our method can accurately detect overlapping pest number 6, in the second column of plots, our method is still able to detect pest number 24 in dense cases, and in the third column of plots, our method detects a larger number of pests compared to other algorithms. It is worth noting that our detector does not detect other noises (pest categories not in our dataset). Experimental results show that the proposed method can improve the detection rate of small and obscured objects.

## 4. Discussion

In this study, we improved the YOLOv3 algorithm with various techniques to achieve optimal performance after 77,900 iterations when the loss was stabilized. Compared with other classical methods, although the second residual block in darknet53 of the proposed method adds two residual units and residual units to replace two DBL units, which increases computational overhead, it can enable the network training deeper, further improve the detection performance of the network and increase the detection rate of pests. We mainly implemented the optimization related to data, anchor generation, and network structure, by cropping and copying the original data for data expansion to reduce the impact of data imbalance on the model. We also scaled the anchors generated by the k-means algorithm using the linear transformation method, and set anchors of different scale sizes for different feature layers, increasing the anchors that match the real labels, which greatly improves the detection accuracy. The output layer removes Branch-1 and Branch-2 which have no role, simplifies the computation of anchor correction during prediction, and improves the detection speed of the model. It can also be seen from Table 6 that the network proposed in this study has the highest mAP, the lowest miss detection rate, and also achieves real-time detection in terms of detection time. Compared with other classical algorithms, the proposed method outperforms other state-of-the-art methods. Of course, although the model can meet the real-time detection requirements, it is still not good enough compared to other lightweight networks and still needs to be improved in terms of mobile applications.

## 5. Conclusion

This study's main goal is to solve the problem of low accuracy of common object detection for small-scale pests, and the challenge focuses on the detection of small size, multi-scale and uneven numbers of pests. Therefore, we proposed an improved method based on YOLOv3, including the cropping and pasting of the real labels of pests, the linear shift of anchors, and the replacement of DBL and Res-Unit in the network of Darknet53 is used to enhance the feature reuse of the model. The experimental results show that the proposed method has improved R and mAP for small targets and effectively improved the detection ability of pests.

In future study, we will focus on the lightweight design of the proposed method by applying Mobilenet, Shufflenet, or other lightweight networks for image feature extraction to reduce the number of parameters without sacrificing model accuracy, and design a more flexible and effective detection filtering method to alleviate the problem of missing detection. In addition, we will expand the pest species and collect more images from actual corn fields to optimize the proposed algorithm.

## Data Availability Statement

The raw data supporting the conclusions of this article will be made available by the authors, without undue reservation.

## Author Contributions

JL designed the study, performed the experiments and wrote the manuscript. WL and CS advised on the design of the model and revised the manuscript. MF collected and analyzed the data. TZ, ZY, and XY supervised and revised the manuscript. SL and CS provided funding support and wrote sections of the manuscript. All the authors contributed to the article and approved the submitted version.

## Funding

This study was supported in part by the Promotion and Innovation of Beijing Academy of Agriculture and Forestry Sciences, Young Beijing Scholars Program, National Natural Science Foundation of China under (Grants 31871525 and 61871475), Beijing Municipal Natural Science Foundation under (Grant 4182023), Laboratory Construction of Guangzhou Innovation Platform Construction Plan under (Grant 201905010006), Guangzhou Key Research and Development Project under (Grants 202103000033 and 201903010043), Guangdong Science and Technology Project under (Grants 2020A1414050060 and 2020B0202080002), Innovation Team Project of Universities in Guangdong Province under (Grant 2021KCXTD019), Guangdong Province Enterprise Science and Technology Commissioner Project under (Grant GDKTP2021004400), Rural Science and Technology Correspondent Project of Zengcheng District, Guangzhou City under (Grant 2021B42121631), and Educational Science Planning Project of Guangdong Province under (Grants 2020GXJK102 and 2018GXJK072).

## Conflict of Interest

The authors declare that the research was conducted in the absence of any commercial or financial relationships that could be construed as a potential conflict of interest.

## Publisher's Note

All claims expressed in this article are solely those of the authors and do not necessarily represent those of their affiliated organizations, or those of the publisher, the editors and the reviewers. Any product that may be evaluated in this article, or claim that may be made by its manufacturer, is not guaranteed or endorsed by the publisher.
