# Supplementary material for: Detecting Pests From Light-Trapping Images Based on Improved YOLOv3 Model and Instance Augmentation
Source: Front Plant Sci. 2022 Jul 7;13:939498. doi: 10.3389/fpls.2022.939498 (PMC9301456; doi:10.3389/fpls.2022.939498)
Supplement: Supplementary file 1 [file Data_Sheet_1.PDF]

Supplementary Material

1 SUPPLEMENTARY FIGURES

Precision-Recall graph showing the curves for the methods shown in Table 5.There are nine pest species in total, and the P-R curves for each model occupy two rows, from top to bottom and left to right, with each figure representing (5, 6, 8, 13, 24, 29, 31, 32, 36)

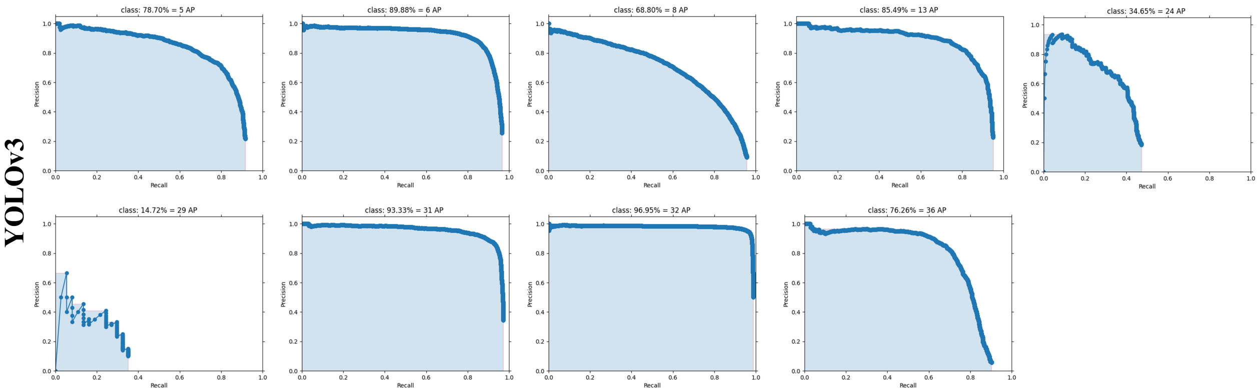

Figure S1. Precision-Recall graph showing the curves for **YOLOv3** shown in Table 5.

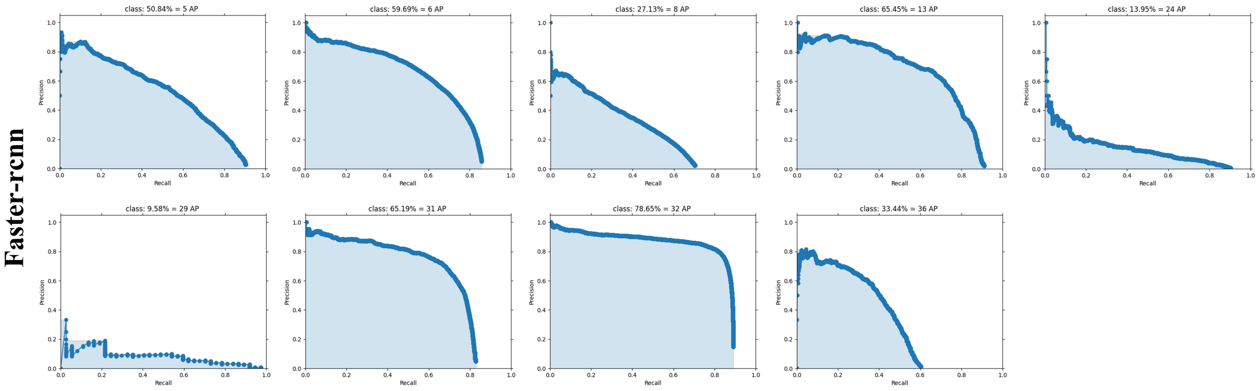

Figure S2. Precision-Recall graph showing the curves for **Faster-rcnn** shown in Table 5.

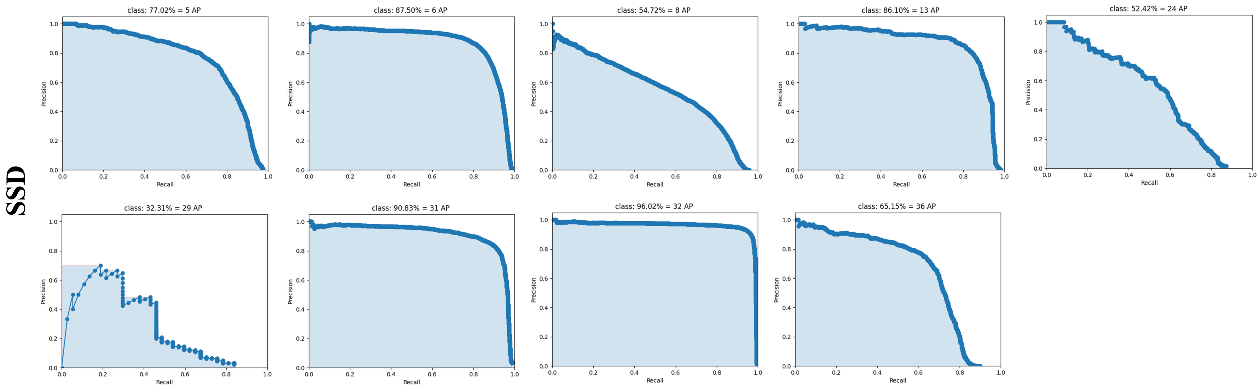

Figure S3. Precision-Recall graph showing the curves for **SSD** shown in Table 5.

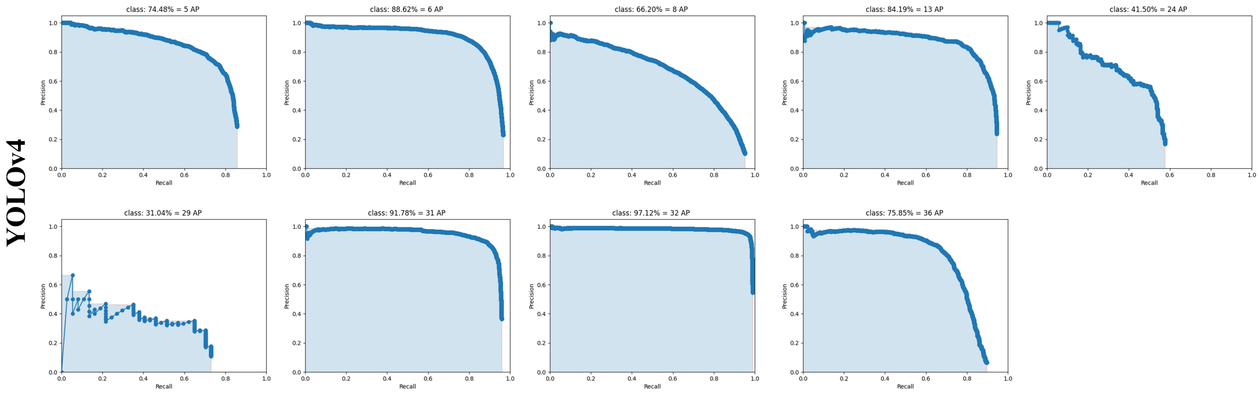

Figure S4. Precision-Recall graph showing the curves for **YOLOv4** shown in Table 5.

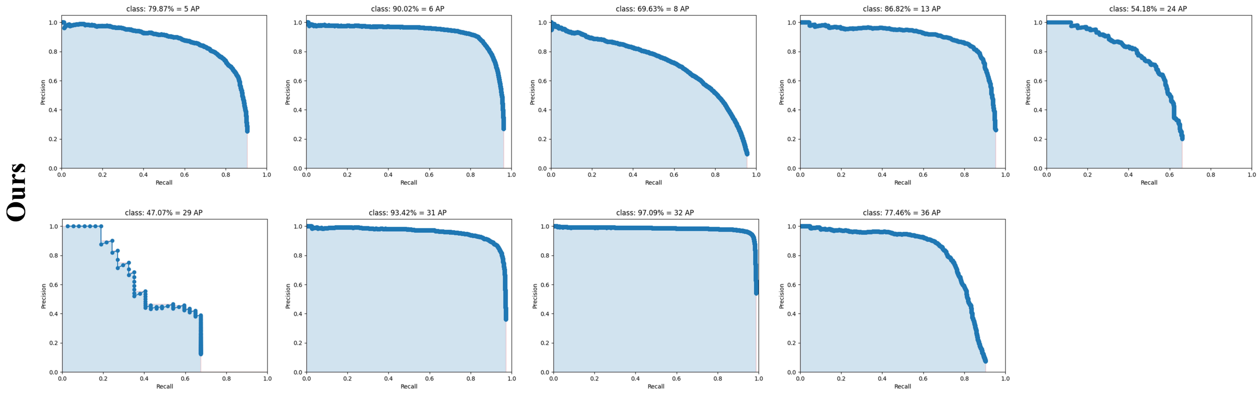

Figure S5. Precision-Recall graph showing the curves for **Ours** shown in Table 5.
